# Water flux patterns and aquaporin dynamics: linking stomatal demand to root hydraulics in pearl millet hybrids

**DOI:** 10.3389/fpls.2026.1776229

**Published:** 2026-04-14

**Authors:** Susan Medina, Aparna Kakkera, Palakolanu Sudhakar Reddy, Vincent Vadez

**Affiliations:** 1 Recuperación Integral de Ecosistemas Naturales y Urbanos (RIENU) Group, Universidad Científica del Sur, Lima, Peru; 2 Crop Physiology Laboratory, International Crops Research Institute for Semi-Arid Tropics, Patancheru, India

**Keywords:** breeding history, drought adaptation, pearl millet, *PIP2;3*, root hydraulics, water use efficiency, xylem anatomy

## Abstract

Water-saving traits are critical for the adaptation of pearl millet (*Pennisetum glaucum*) to arid environments. This study investigates how breeding history and specifically selection in High Rainfall (HR) versus Low Rainfall (LR) zones influences root hydraulics and the molecular regulation of aquaporins (AQP). The study utilized four contrasting hybrids to integrate physiological assays with anatomical and molecular phenotyping. Key methodologies included measuring transpiration responses to vapor pressure deficit (VPD), root pressurization, and pharmacological inhibition. These were coupled with anatomical characterization of root systems, aerial growth and transcriptional profiling of *PIP* and *TIP* (Plasma membrane/Tonoplast Intrinsic Protein) genes. LR hybrids exhibited an "opportunistic" strategy characterized by superior root and canopy growth supported by extensive metaxylem proliferation. However, these genotypes showed lower constitutive root hydraulic conductivity and a steeper transpiration increase during pressurization, suggesting a radial transport bottleneck. At the molecular level, HR hybrids significantly downregulated *PIP2;3* in roots under high VPD. In contrast, LR hybrids maintained *PIP2;3* expression, facilitating radial flow to match their high transpiration demand. The findings demonstrate that breeding for specific rainfall zones has shaped divergent hydraulic strategies. HR genotypes utilize a conservative "hydraulic brake" mechanism driven by AQP downregulation to conserve water. Conversely, LR genotypes employ an acquisitive strategy, relying on coordinated anatomical capacity and molecular gating to meet high transpiration demands. These distinct mechanisms provide a blueprint for selecting resilient crops based on specific environmental pressures.

## 1 Introduction

Pearl millet (*Pennisetum glaucum* L.) stands as a mainstay of food security in the Sahel and India, where it ranks second in agro-economic importance only to sorghum due to its exceptional adaptation to arid environments (Manga and Kumar, 2011; Rai et al., 2015). However, the escalating challenges of global climate change, driven by rising temperatures and water scarcity, necessitate a mechanistic understanding of Water Use Efficiency (WUE) and adaptation strategies in such resilient crops (Kholová et al., 2010; Kurowska, 2021; Shrestha et al., 2023). Specifically, dissecting the physiological and molecular basis of the transpiration response to increasing VPD is critical to ensuring future crop stability (Vadez et al., 2013).

Historically, the All India Coordinated Pearl Millet Improvement Project (AICPMIP) stratified cultivation into distinct agro-climatic zones based on precipitation patterns and soil typology (Manga and Kumar, 2011; Rai et al., 2015). These zones (**Supplementary Figure 1**) present sharply contrasting selective pressures: the arid Zone A1 in Northwestern India is characterized by sandy Entisols and low rainfall (320–400 mm), whereas the semi-arid Zone B in Peninsular India features heavy Alfisols and higher precipitation (400–520 mm) (Vadez et al., 2015; Medina et al., 2017 and Garin et al., 2023). Breeding selection within these divergent environments has driven the evolution of distinct water-use strategies. Genotypes adapted to the erratic moisture of Zone A1 typically prioritize rapid water uptake via high tillering capacity and reduced leaf area to mitigate evaporative demand (Srivastava et al., 2022; Shrestha et al., 2023; Grégoire et al., 2024). Conversely, HR hybrids from the wetter Zone B tend to develop larger canopies to maximize light interception, relying instead on stomatal closure under high VPD to regulate plant water status (Medina et al., 2017). This specific adaptation history provides a unique genetic framework to dissect the physiological mechanisms of drought tolerance.

Central to these adaptive mechanisms are Aquaporins (AQPs), members of the major intrinsic protein (MIP) family. These integral membrane proteins form passive pores (Kruse et al., 2006) that are tightly regulated by gating mechanisms, such as phosphorylation and pH changes, which modulate water permeability in response to environmental changes (Patel and Mishra, 2021; Shivaraj et al., 2021; Maurel et al., 2015; Kapilan et al., 2018). AQPs facilitate the transport of water and small neutral molecules across biological membranes, which is a critical process for plant growth (Li et al., 2014; Maurel et al., 2015), for example PIP2 is important in rice and grapevine (Bai et al., 2021; Vandeleur et al., 2008). Consequently, they play a pivotal role in controlling root hydraulics and leaf gas exchange (Forrest and Bhave, 2007; Grondin et al., 2020) such as CO_2_ diffusion, and also for abiotic-stress tolerance such as drought, heat, salinity and nutrient limitation (Wang et al., 2020; Pawłowicz and Masajada, 2019; Patel and Mishra, 2021; Deshmukh et al., 2017; Kurowska, 2021). Aquaporin are also important for xylem conductance like in walnut where they may be controlling solute flux to the parenchyma cells (Sakr et al., 2003) and the transport of metalloids like boric or silicic acid (Kaldenhoff et al., 2008; Li et al., 2014), or like in rice and mungbean (Van Den Berg et al., 2021; Thakral et al., 2023).

Among the diverse AQP subfamilies, plasma membrane intrinsic proteins (PIPs) and tonoplast intrinsic proteins (TIPs) are of particular interest due to their abundance in the primary transcellular and intracellular water transport pathways in roots, stems, and leaves (Kapilan et al., 2018; Shivaraj et al., 2021; Sudhakaran et al., 2021; Hewawitharanage and Sarvananda, 2024). In drought-adapted cereals like pearl millet, specific isoforms exhibit specialized roles in hydraulic regulation under stress (Maurel, 2007). For instance, the transcript abundance of *PIP2;3* has been correlated with high transpiration rates under high VPD, suggesting a role in facilitating water transport during peak evaporative demand (Reddy et al., 2017). Conversely, *PIP2;6* appears to function in water conservation; its overexpression results in reduced transpiration and improved abiotic stress tolerance (Reddy et al., 2022). Furthermore, while TIPs are traditionally localized in the vacuolar membrane, the pearl millet isoform *TIP2;2* also can be predicted to localize, *in silico*, in the plasma membrane, where they may be playing a potentially novel role in osmotic adjustment (Reddy et al., 2017, Reddy et al., 2022) although definitive experimental localization has not yet been reported.

Root hydraulics modulate water uptake through a dynamic balance between the apoplastic pathway (predominant under high transpiration for coarse regulation) and the cell-to-cell pathway, which allows for fine hydraulic tuning via aquaporins (Maurel et al., 2010; Vadez, 2014). Notably, genotypes exhibiting high WUE often show reduced dependence on AQP-mediated transport compared to low-WUE lines, implying a conservative hydraulic strategy (Grondin et al., 2020). This adaptive “hydraulic braking” is achieved through the downregulation of specific isoforms, such as *PIP1;3* and *PIP2;6*, effectively limiting radial water flow during stress (Grondin et al., 2020; Reddy et al., 2022).

While root system architecture (RSA) traits, particularly rooting depth and root length density (RLD), remain fundamental for deep soil water extraction (Vadez, 2014; Shrestha et al., 2023), RSA alone implies a correlation rather than a complete mechanism for yield stability. Fine anatomical traits are equally critical regulators of hydraulic flow (Maurel and Nacry, 2020). Although metaxylem (geometry of single xylem vessel in the seminal root axes) restriction was successfully exploited to improve drought tolerance in wheat (Richards, 2006), recent insights in pearl millet suggest a more complex coordination. High WUE lines potentially compensate for reduced AQP activity via structural optimizations, such as altered endodermal barriers or expanded late metaxylem vessels (Kholová et al., 2016; Lucas Gutiérrez, 2025). These features balance radial and axial hydraulic conductance, explaining the restriction of transpiration under high VPD (Srivastava et al., 2022). Specifically, narrowing the metaxylem vessel diameter supports a conservative strategy, which limits axial conductance and transpiration while enhancing cavitation resistance to prevent hydraulic failure (Barry et al., 2025).

To dissect these complex hydraulic properties, from water fluxes to tissue-level conductivity, studies frequently employ pharmacological inhibitors such as mercuric chloride (HgCl_2_) and silver nitrate (AgNO_3_). These agents block AQP channels via steric hindrance or binding to sulfhydryl groups, thereby altering tissue hydraulic conductivity and allowing for the quantification of AQP-mediated transport (Niemietz and Tyerman, 2002; Nardini and Salleo, 2005; Savage and Stroud, 2007).

Therefore, we hypothesized that drought adaptation in pearl millet relies on the functional coordination between root anatomy and molecular regulation, shaped by the selective pressures of India’s agro-climatic zones. We proposed that HR genotypes use *PIP2;3* downregulation as a “conservative” hydraulic brake, whereas LR genotypes maintain *PIP2;3* expression to support an “opportunistic” surge in radial flow towards the metaxylem. This hypothesis suggests that differential aquaporin regulation allows dry-zone hybrids to overcome radial resistance and maximize transpiration when water is available. The objective of this study was to elucidate the mechanistic coupling between stomatal water-saving traits and root hydraulic properties in pearl millet. We specifically compared hybrids adapted to contrasting rainfall zones (zone A1 vs. zone B) by integrating physiological assays, quantifying the contribution of aquaporin-mediated pathways to root hydraulic conductivity and transpiration responses under pressurization, with the molecular profiling of aquaporin transcript abundance under high Vapor Pressure Deficit.

## 2 Material and methods

Four pearl millet (*Pennisetum glaucum* L.) hybrids bred for contrasting agro-ecological zones in India were evaluated. Two genotypes originated from the HR zone B (HR: AHT-II/K13–24 and AHT-II/K13-5), and two were developed for the arid, LR zone A1 (LR: HOPE 2013-AHT-R-14 and HOPE 2013-AHT-R-8). The physiological and morphological traits of these hybrids were characterized across four distinct experiments (Exp. 1–4). Exp. 1 assessed the contribution of the cell-to-cell pathway to water transport by measuring transpiration rate responses (TR) to aquaporin inhibition (pharmacological treatments) in hydroponically grown plants under high evaporative demand. Exp. 2 investigated potential root hydraulic limitations by analyzing TR responses to root pressurization under similar high evaporative demands. Exp. 3 elucidated the molecular regulation of water flow by profiling the transcript abundance of three specific aquaporin genes in roots and shoots. Finally, Exp. 4 monitored canopy development and leaf expansion dynamics using the high-throughput LeasyScan platform (Vadez et al., 2015). Experiments 1 (n=60, 15 plants per genotype), 2 (n=80, 20 plants per genotype), 3 (n=80, 20 plants per genotype), 4 (n=24, 6 plants per genotype) were all conducted at the vegetative stage using a completely randomized design with five to ten biological replicates per genotype in each treatment. All studies (were carried out under controlled conditions between February and April 2016 at the ICRISAT campus in Patancheru, India (17°30´N; 78°16´E; altitude 549 m).

### 2.1 Experiment 1: transpiration response to aquaporin inhibition under high evaporative demand

This experiment quantified the impact of aquaporin inhibition on transpiration rates in pearl millet hybrids with contrasting responses to VPD. Sixty plants (n=60) were grown (15 plants per genotype, 5 replicates as controls, 5 replicates to test mercuric chloride (HgCl_2_) inhibitor and 5 replicates to test silver nitrate (AgNO_3_) inhibitor) during February–March 2016 under controlled hydroponic glasshouse conditions (17–35 °C; 35–70% RH). Seeds were germinated for 7 days in moist sand using Hoagland**’**s nutrient solution (Hoagland and Arnon, 1950) prepared in deionized water. Seedlings were subsequently transferred to a hydroponic system consisting of 500 ml flasks filled with nutrient solution and subjected to constant aeration (0.5 kPa). The solution was topped up daily and completely replaced every third day.

At 25 days after sowing (DAS), the transpiration response (TR) to aquaporin inhibitors was assessed under high VPD. Plants were transferred to a Conviron E-15 growth chamber (Controlled Environments, Winnipeg, MB, Canada) for overnight acclimation at a low VPD of 0.5 kPa (23 °C; 80% RH) with continuous aeration. On the assay day, light intensity was ramped up starting at 06:00 h, reaching a constant Photosynthetic Photon Flux Density (PPFD) of ~400 µmol m^-2^s^-1^ by 08:30 h. Concurrently, VPD was progressively increased to reach a high evaporative demand target of 3.0 kPa (36 °C; 55% RH) at 08:30 h, which was maintained throughout the experiment.

The assay consisted in measuring transpiration over three consecutive 2-hour phases: (i) Baseline (08:30–10:30 h), (ii) Inhibition (10:30–12:30 h), and (iii) Recovery (12:30–14:30 h) (Tharanya et al., 2018). Transpiration was gravimetrically recorded every 20 min. At the end of the baseline period, aquaporin inhibitors were introduced by adding 500 *μl* of stock solutions to the nutrient medium to reach final concentrations of 100 *μM* HgCl_2_ and 10 *μM* AgNO_3_. For the recovery phase, the medium in all flasks was replaced with fresh, inhibitor-free nutrient solution. Control plants underwent the same handling without inhibitor addition. Upon completion of the assay, stems were cut, and xylem exudates were collected for 20 min into pre-weighed tubes containing absorbent paper. Subsequently, leaves and roots were harvested. Leaf area was quantified using a leaf area meter (Model LI-3000, Li-Cor, Lincoln, NE, USA). Roots were rinsed with deionized water and scanned using the WinRHIZO™ Pro system (Regent Instruments Inc., Quebec City, Canada). Finally, dry weights were determined for all organs after drying at 60 °C for 72 h.

### 2.2 Experiment 2: transpiration response to root pressurization and hydraulic conductivity assessment

This experiment quantified root hydraulic limitations by measuring transpiration increases in response to root pressurization. Eighty plants (n=80, 20 plants per genotype considering 10 replicates as controls plus 10 replicates as pressurized plants per each genotype) were grown during March 2016 under controlled glasshouse conditions (18–35 °C; 35–70% RH). Seeds were sown in cylindrical polystyrene bags containing 1.5 kg of a Vertisol:sand mixture (1:1 v/v) to shield roots from light. Seedlings were fertigated every third day with 50% strength Hoagland**’**s solution (as described in Exp. 1).

Pressurization assay setup - At 30 DAS, half of the plants (treated group) were transferred into pressure chambers pots similar to those described by Choudhary and Sinclair (2014), involving the removal of the plastic bag and backfilling the pot with soil while minimizing root disturbance. These plants were watered and acclimated with open lids for two days. The remaining plants (control group, which was not subjected to pressure) were transferred to similar pots whose soil surface was covered with a polystyrene sheet and plastic beads to prevent soil evaporation.

Transpiration response to pressure – At 33 DAS in the morning, the pots were sealed. Both control and pressurized pots were weighed every 2 h using an electronic balance (FBK, Kern & Sohn GmbH, Balingen, Germany) to determine basal transpiration rates. Following the initial 2-hour baseline, positive pressure was applied to the root systems to increase leaf xylem hydrostatic water potential (Sinclair et al., 2008). Pressure was applied in two sequential 2-hour steps: 0.15 MPa and 0.25 MPa. Transpiration rates (TR) were calculated gravimetrically for each period. To estimate the hydraulic limitation of whole-plant conductance, the TR of pressurized plants was normalized against the TR of control plants under ambient conditions. Leaf area was measured post-harvest (Model LI-3100, Li-Cor, Lincoln, NE, USA) to express TR per unit leaf area.

Root hydraulic conductivity (Lpr) and anatomy - Immediately following the transpiration assay (30 min after the last pressure step), shoots were excised leaving a ~2 cm stem stump. Pre-weighed vials containing absorbent cotton were attached to the stump to collect xylem exudate. In treated plants, exudation was induced by applying three consecutive pressure levels (0.05, 0.15, and 0.25 MPa) for 10 min each. In control plants, spontaneous exudate was collected at ambient pressure for 10 min. Xylem flux was determined gravimetrically.

Roots were subsequently washed and scanned using WinRHIZO™ software to measure total root length (RL) and root surface area (RSA). Root hydraulic conductivity (Lpr) was calculated as the slope of the flow rate normalized by RL and RSA versus the driving force (pressure). For anatomical analysis, 2 cm segments were excised from the mid-section of the longest roots and stored in saline solution (0.85% NaCl) to keep root tissues isotonic and structurally intact for sectioning and to avoid vessel collapse (Miloud et al., 2020; Zhang et al., 2025). Cross-sections were stained with 5% Acid Fuchsin and imaged using light microscopy at 10X magnification. Image analysis was performed using ImageJ software (NIH, Bethesda, MD, USA). Dry weights of all organs were determined as described in Exp. 1.

### 2.3 Experiment 3: transpiration response to evaporative demand and aquaporin gene expression

This experiment investigated the transcriptional regulation of aquaporin genes in pearl millet genotypes with contrasting transpiration responses to high VPD. Eighty plants (n=80) were grown during February–March 2016 under glasshouse conditions as described in Exp. 2, but using 8 kg capacity pots filled with sand. Plants were fertigated every two days with nutrient solution (see Exp. 1).

VPD treatment and physiological assay - At 30 DAS (6-leaf stage), plants were fully irrigated, sealed with plastic sheets and beads to prevent soil evaporation, and allowed to drain overnight. The following day, pots were transferred to two Conviron E-15 growth chambers (Controlled Environments, Winnipeg, MB, Canada) for overnight acclimation at a low VPD of 0.5 kPa (23 °C; 80% RH). On the assay day, one chamber was maintained at a constant low VPD (1.0 kPa; 25 °C; 70% RH) to serve as a control. The second chamber subjected plants to a stepwise VPD gradient ranging from 1.0 to 4.0 kPa, which was achieved by modulating temperature and humidity from 25 °C/70% RH to 40 °C/45% RH. Transpiration was recorded gravimetrically using an electronic balance (FBK, Kern & Sohn GmbH, Balingen, Germany) from 07:30 h to 16:00 h. The initial period (07:30–08:30 h; VPD 1.0 kPa) served as the baseline. Transpiration responses (TR) under the increasing VPD gradient were normalized against transpiration of plants in the constant VPD chamber.

Tissue sampling for gene expression was synchronized with specific VPD thresholds: The baseline control (C) was sampled at 08:30 h (1.0 kPa) from both chambers. T1 was the low VPD control, sampled at 15:45 h from the constant low VPD chamber. T2 was the high VPD treatment sampled at 15:50 h from the high VPD chamber (peak stress). At each time point, the central portion of the last fully developed leaf and a pooled sample of the central root system (washed with deionized water) were harvested, immediately frozen in liquid nitrogen, and stored at -80 °C. Leaf area and dry weights were determined post-harvest as previously described.

RNA extraction and quantitative real-time PCR (qRT-PCR) - Frozen root and leaf tissues (n=160: 80 from C, 40 from T1, and 40 from T2) were ground in liquid nitrogen. Total RNA was isolated from 100 mg of tissue using the NucleoSpin^®^ RNA Plant Kit (Macherey-Nagel GmbH & Co. KG, Düren, Germany), including on-column DNase digestion. RNA integrity was verified via 1.2% agarose gel electrophoresis, and concentration was quantified using the Qubit^®^ HS RNA Assay Kit (Thermo Fisher Scientific Inc., MA, USA). Samples were stored at -70 °C.

First-strand cDNA was synthesized from 500 ng of total RNA using M-MuLV reverse transcriptase, oligo d(T)23VN, and RNase inhibitor M0314S (New England Biolabs Inc., MA, USA) following the manufacturer’s instructions. The cDNA was diluted to 10 ng. µl^-1^ and stored at -20 °C. Three housekeeping genes (EIF4α, EF-1α and ACP) and three target aquaporin genes (*PIP2;3*, *PIP2;6*, and *TIP2;2*) were analyzed using specific primers (**Supplementary Table 1**), the efficiency of which was validated in previous studies (Reddy et al., 2015a, 2015b).

qRT-PCR assays were performed using a Realplex Real-Time PCR system (Eppendorf, Germany) and SYBR Green chemistry in 96 optical-well-plates (Axygen, USA) sealed with ultra-clear sealing film (Platemax). Reactions (10 µl total volume) contained 5 µl of 2x SensiMix SYBR No-ROX kit (Bioline, London, UK), 400 nM of each primer, 1 µl of diluted cDNA (1:10), and nuclease-free water. The thermal profile consisted of an initial denaturation at 95 °C for 2 min, followed by 40 cycles of 95 °C for 15 s and 62 °C for 30 s (data acquisition of fluorescent signal), ending with a melt curve analysis to verify specificity. All reactions, including non-template controls, were performed in technical triplicates. Relative expression was analyzed using the comparative C_t_ method. Expression ratios were calculated as 2^-ΔCt^ (log_2_ transformed), and fold changes between treatments (C, T1, T2) were determined using the 2^-ΔΔCt^ method (Schmittgen and Livak, 2008). Internal control genes were used for normalization as established in previous reports (Reddy et al., 2015a, 2015b).

### 2.4 Experiment 4: canopy growth under high VPD conditions (LeasyScan)

This outdoor experiment measured putative genotypic differences in aerial development under natural high evaporative demand and tested the hypothesis that these differences are linked to root water transport capacity. The trial was conducted during the dry, high-temperature season (March–April 2016) using the LeasyScan high-throughput phenotyping platform (Vadez et al., 2015). The experiment coincided with a period of high atmospheric demand (high VPD ~5 kPa). Temperatures fluctuated between 16 °C and 41 °C, and relative humidity percentage (RH%) ranged from 12% to 87%. Except for a single rainfall event (5 mm), VPD remained high, ranging daily from 1.0 to 6.5 kPa. Six biological replicates were evaluated for each genotype (n=24 total).

Experimental setup and sensing - Seeds were sown in 15 kg capacity pots filled with Alfisol. At 12 DAS, seedlings were thinned to two plants per pot and grown for a total of 48 days. Pots were automatically irrigated every 1–3 days with fresh water to ensure non-limiting moisture. The LeasyScan platform utilizes laser triangulation to generate 3D point clouds of the canopy. Plant parameters (leaf area, plant height) and environmental data were captured every 2 hours using PlantEye F500 sensors (Phenospex, Heerlen, The Netherlands).

Data processing and modeling - To account for thermal fluctuations affecting development, daily growth rates were normalized to equivalent days at 20 °C following the thermal time model of Parent and Tardieu (2012). Scanned 3D leaf area (LA3d) was corrected to observed leaf area (LA) using the validated transformation equation reported by Vadez et al. (2015) ( LA3d = 0.22 LA + 241). Canopy growth kinetics were modeled using non-linear sigmoidal regression. To compare growth vigor between rainfall zones, the slope of the linear phase (exponential growth) was extracted and analyzed.

### 2.5 Data analysis and statistical methods

Data analysis was tailored to the specific design of each experiment to quantify physiological responses accurately. Exp. 1 (Inhibitor Response): To quantify the effect of aquaporin inhibitors, TR of treated plants was normalized against non-treated controls to obtain the normalized transpiration rate (NTR). The aggregate response to inhibition was analyzed by calculating the area under the curve (AUC) from the NTR vs. time plot using an iterative integration method (1000 iterations). Exp. 2 (Hydraulics): Root hydraulic conductivity was derived as the slope of the linear regression between xylem exudate flow normalized by root length, or by root surface area, and the driving force (applied pressure). Similarly, whole-plant conductance limitation was assessed by normalizing the TR of pressurized plants against controls. Exp. 3 (transpiration response to VPD and AQP transcription): Transpiration data under the VPD gradient were normalized to the baseline TR at constant low VPD. The response was fitted using a segmented linear regression model (bilinear model) to identify breakpoints. The model consists of two intersecting linear segments: Y_1_ = slope_1_.X + intercept_1_ (before breakpoint) and Y_2_ = slope_2_.X + intercept_2_ (after breakpoint), fitted with 1000 iterations. For gene expression analysis, relative expression values calculated via the comparative C_t_ method were log_2_ transformed to ensure normal distribution prior to analysis. Exp. 4 (Canopy Growth): Leaf area expansion was modeled using sigmoidal nonlinear regression.

To compare the physiological and molecular traits between LR and HR genotypes, data from all experiments were subjected to analysis of variance (ANOVA). *Post-hoc* mean comparisons were performed using Fisher’s least significant difference (LSD) test. All measured traits were subjected to a Pearson correlation analysis. Statistical significance was set at p< 0.05. Letters in figures denote significant differences based on the LSD test. All AUC analyses, linear/nonlinear regressions, correlations and curve fittings were performed using GraphPad Prism (Version 7.00, GraphPad Software, La Jolla, CA, USA). ANOVA and LSD tests were conducted using the R software environment (R Development Core Team, 2023).

## 3 Results

### 3.1 Inhibition of transpiration response

**Figure 1:**
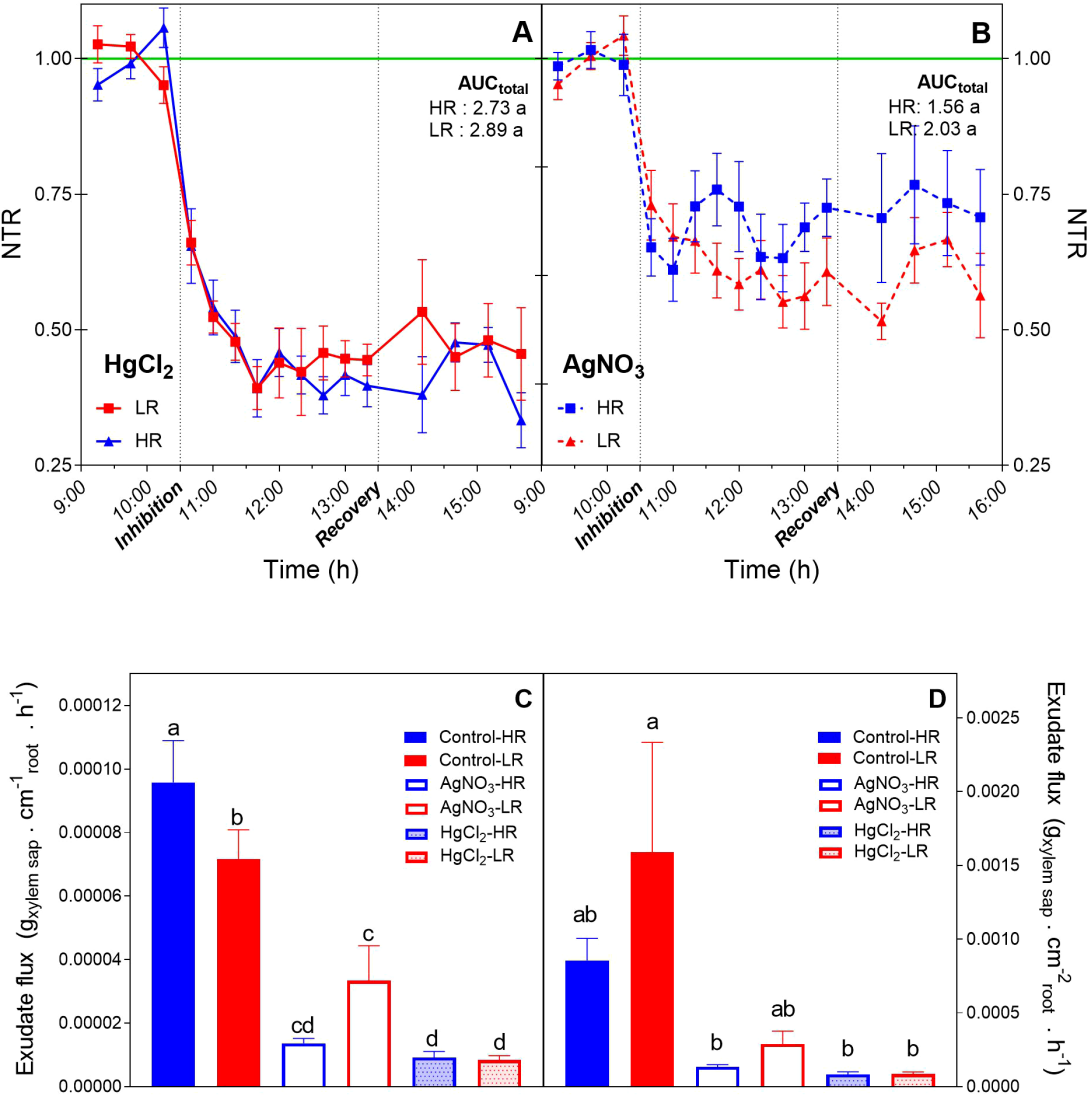
Effect of pharmacological aquaporin inhibition on whole-plant transpiration and root hydraulic flux in pearl millet hybrids. **(A, B)** Normalized Transpiration Rate (NTR) kinetics following treatment with the aquaporin blockers **(A)** mercuric chloride (HgCl_2_) and **(B)** silver nitrate (AgNO_3_). The magnitude of inhibition relative to the non-treated plants baseline (green line) was quantified using the area under the curve (AUC) analysis. Lpr is shown as xylem exudate flux normalized to **(C)** total root length (RL) and **(D)** root surface area (RSA) under control conditions and following inhibitor application. Data represent means (of 5 replicates per treatment and genotype) of high rainfall (HR) and low rainfall (LR) genotypes. Different letters indicate significant differences between genotypes and treatments according to Fisher’s LSD test (p< 0.05).

Pharmacological blockage of aquaporins significantly reduced transpiration rates (TR) in all four hybrids tested (**Supplementary Figure 2**). Notably, this inhibition appeared irreversible within the experimental timeframe, as TR failed to recover following the replacement of the inhibitor solution with fresh nutrient medium. The magnitude of restriction varied by compound: mercuric chloride (HgCl_2_) induced a severe NTR reduction, ranging from 25% to 50% relative to controls (**Figure 1A**). In contrast, silver nitrate (AgNO_3_) caused a milder NTR decrease of approximately 25% across all genotypes (**Figure 1B**). Analysis of the AUC revealed no significant differences between LR and HR groups regarding the extent of whole-plant transpiration inhibition, although HR hybrids displayed a slight, non-significant trend toward lower sensitivity to AgNO_3_.

While basal transpiration rates were comparable between rainfall groups during the control period (**Figures 1A, B**), direct measurements of root hydraulics revealed distinct genotypic patterns. Under control conditions, Lpr of HR hybrids exhibited a significantly higher specific xylem mass flow (normalized to root length) compared to LR hybrids (**Figure 1C**) while there were no significant differences between both groups when it was normalized by root surface area (**Figure 1D**). Both inhibitors reduced xylem flux, with HgCl_2_ exerting the most potent effect. Interestingly, HR hybrids appeared more sensitive to this blockade than LR lines in terms of root exudation, whereas AgNO_3_ treatment did not result in statistically significant differences in exudate flow between the groups.

### 3.2 Hydraulic conductance limitations

**Figure 2:**
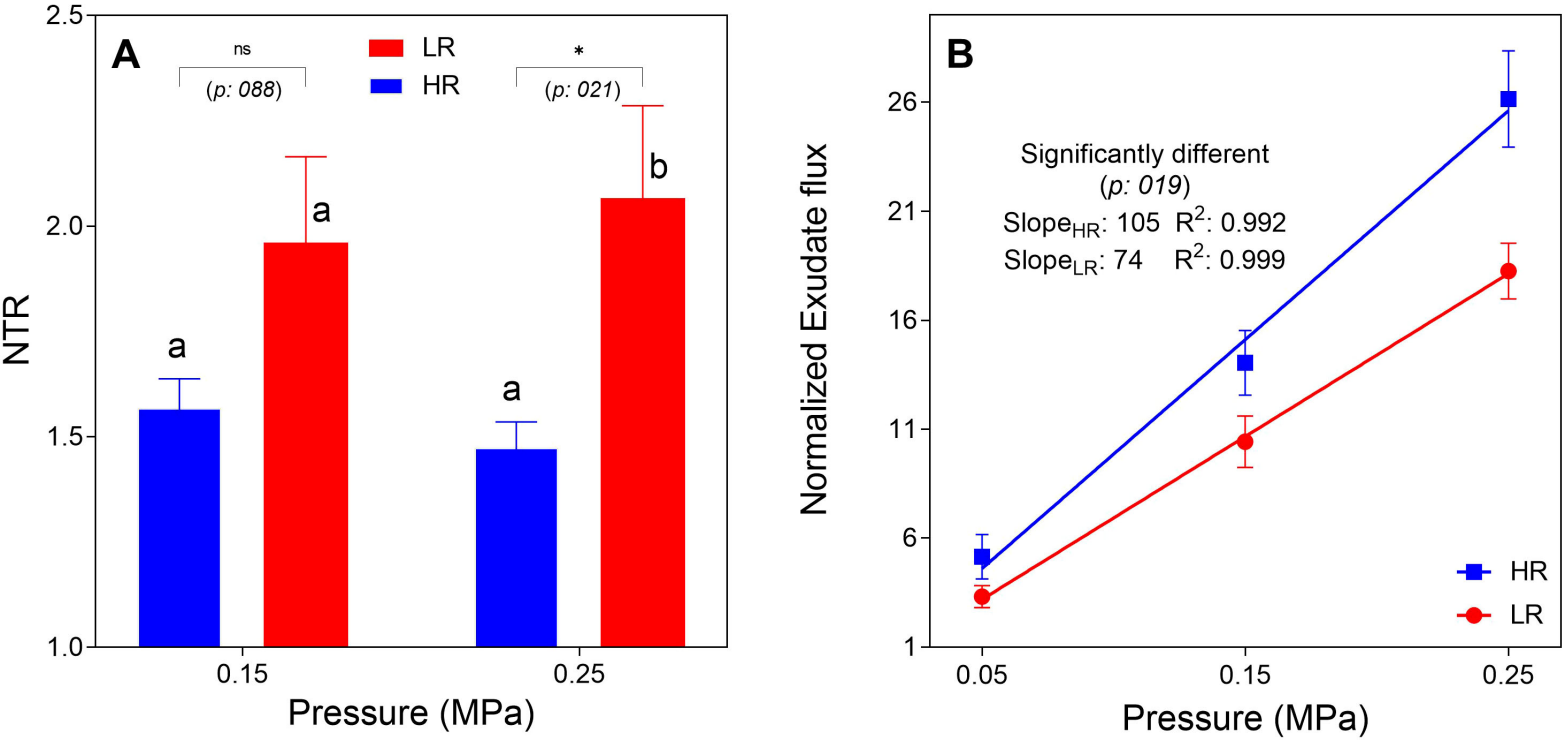
Whole-plant transpiration response to pressurization and root hydraulic conductivity in pearl millet hybrids. **(A)** Assessment of whole-plant hydraulic limitation. Data show the normalized transpiration rate (NTR) of high rainfall (HR) and low rainfall (LR) genotypes in response to applied root pressure (0.15 and 0.25 MPa). Higher NTR values indicate greater relief from hydraulic constraints. Letters denote significant differences between genotypic groups according to Fisher’s LSD test (p< 0.05). **(B)** Determination of root hydraulic conductivity. The plot displays specific xylem flux normalized by root length as a function of the driving pressure gradient (0.05, 0.15, and 0.25 MPa). It corresponds to the slope of the linear regression for each group. The slopes of HR and LR hybrids were compared using an F-test (p< 0.05).

Application of pressure to the root system resulted in a marked increase in transpiration across all genotypes, confirming the existence of hydraulic limitations under ambient conditions. The NTR, expressing the proportional transpiration increase relative to non-pressurized controls, rose by approximately 50% to 100% depending on the pressure intensity (**Figure 2A**). Notably, hybrids from the LR zone exhibited a significantly stronger response to pressurization than HR hybrids, particularly at the highest-pressure step (0.25 MPa). A similar trend was observed at the lower pressure step (0.15 MPa), although the genotypic differences were not statistically significant at this level. Following the whole-plant assay, root hydraulic conductivity was quantified directly by measuring xylem exudate flow rates in response to increasing pressure gradients (**Figure 2B**). Consistent with the transpiration data, it was significantly lower in LR hybrids (Slope = 74 g cm^-1^ h^-1^ MPa^-1^) compared to HR hybrids (Slope = 105 g cm^-1^ h^-1^ MPa^-1^), indicating a higher constitutive hydraulic resistance in the drought-adapted genotypes.

### 3.3 Transpiration dynamics and aquaporin gene expression under high VPD

**Figure 3:**
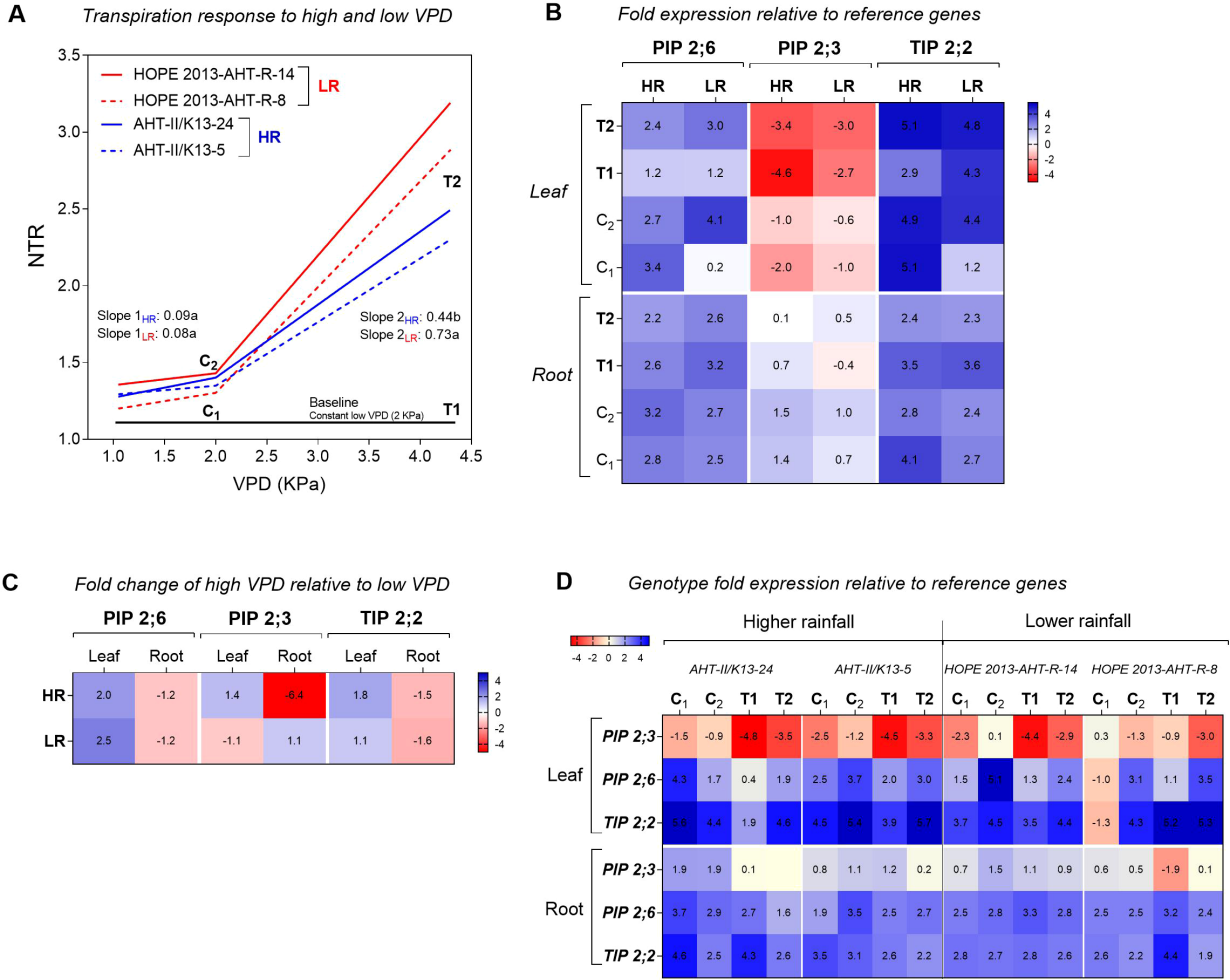
Transpiration kinetics and aquaporin transcriptional profiling under varying evaporative demand. **(A)** Normalized transpiration rate (NTR) response to increasing vapor pressure deficit (VPD). Data show the transpiration of high rainfall (HR) and low rainfall (LR) hybrids subjected to a stepwise VPD gradient (from morning-C_1_ and C_2_ 2kPa to afternoon-T2 peak 4.0 kPa), normalized against controls maintained at constant low VPD (T1; 1.0 kPa). Curves represent the fitted segmental linear regression. Differences in slopes between rainfall groups were significant (p< 0.05). **(B)** Relative transcript abundance of *PIP2;3*, *PIP2;6*, and *TIP2;2* in leaf and root tissues. Expression levels are normalized to internal reference genes and presented as *log_2_(2^-ΔCt^*). Sampling points correspond to: morning baseline (C_1_ and C_2_), afternoon low VPD control (T1), and afternoon high VPD Stress (T2). **(C)** Transcriptional regulation under high evaporative demand. Data represent the *log_2_* fold change *(2^-ΔΔCt^*) of transcript abundance under high VPD (T2) relative to the low VPD control (T1) at the same time point. Positive values indicate upregulation; Negative values indicate downregulation. **(D)** Genotypic variation in aquaporin expression in leaf and root tissues, quantified by real-time PCR, in relation to transpiration responses under low (T1; 1 kPa) and high (T2; 1 to 4.5 kPa) VPD conditions, as well as in control low VPD sampling points (C1 and C2). The heatmap represents log-transformed real-time PCR data expressed as ΔCt (C_T_ mRNA - C_T_ Reference RNA). Detailed statistical comparisons are provided in **Supplementary Table 2**.

To isolate the VPD effect, the transpiration response of hybrids subjected to a VPD gradient (morning-C to afternoon-T2) was normalized against controls maintained at constant low VPD (**Figure 3A**). NTR revealed distinct sensitivity patterns. While both groups exhibited similar baseline slopes at low VPD (0.08–0.09 kPa), LR hybrids displayed a significantly steeper increase in transpiration in response to rising VPD (Slope = 0.73) compared to HR hybrids (Slope = 0.44). This suggests a more conservative stomatal regulation in HR hybrids under high evaporative demand.

The gene expression analysis via qRT-PCR revealed tissue-specific and genotype-dependent regulatory patterns (**Figure 3B**). Generally, *PIP2;6* and *TIP2;2* transcript abundances were upregulated relative to housekeeping genes in both tissues, with *TIP2;2* showing particularly high expression levels. In contrast, *PIP2;3* displayed a divergent pattern, being notably downregulated in the roots of HR hybrids.

The specific transcriptional response to high VPD stress (fold change between the high VPD-T2 samples versus the low VPD-T1 samples) highlighted crucial adaptive strategies (**Figure 3C**) and a variation within genotypes adapted to LR and HR zones (**Figure 3D**). In leaves, the high VPD treatment triggered a generalized upregulation of AQPs. *PIP2;6* expression increased approximately 2-fold across all genotypes. Similarly, *TIP2;2* was upregulated (~1-fold), with a stronger response in HR hybrids. For *PIP2;3*, HR hybrids showed a 1.4-fold induction, whereas LR hybrids exhibited a slight downregulation (1.1-fold). In contrast to leaves, roots generally exhibited downregulation under high VPD. *PIP2;6* and *TIP2;2* were repressed (1.2-fold and 1.5-fold reduction, respectively) across both groups. The most striking genotypic difference occurred in root *PIP2;3* regulation. While LR hybrids maintained stable or slightly increased expression (1.1-fold upregulation), HR hybrids exhibited a drastic downregulation, reducing transcript abundance by 6-fold. This massive repression in HR roots coincides with their lower hydraulic slope observed in the physiological assay. In leaves, both (HR) genotypes exhibited a downregulation of *PIP2;3* from morning to afternoon under high VPD conditions (T2). Furthermore, under constant low VPD (T1), its expression was 4.5- to 4.8-fold lower. In contrast, LR genotypes displayed variable expression patterns. In roots, *PIP2;3* was similarly downregulated from morning to afternoon under high VPD in both HR genotypes, whereas LR genotypes maintained stable expression levels. Regarding *PIP2;6*, expression patterns were consistent across all four genotypes in both leaves and roots. Finally, *TIP2;2* showed stronger upregulation in leaves compared to roots, with only minor variations among the four genotypes.

### 3.4 Root architecture, anatomy, and canopy development

**Figure 4:**
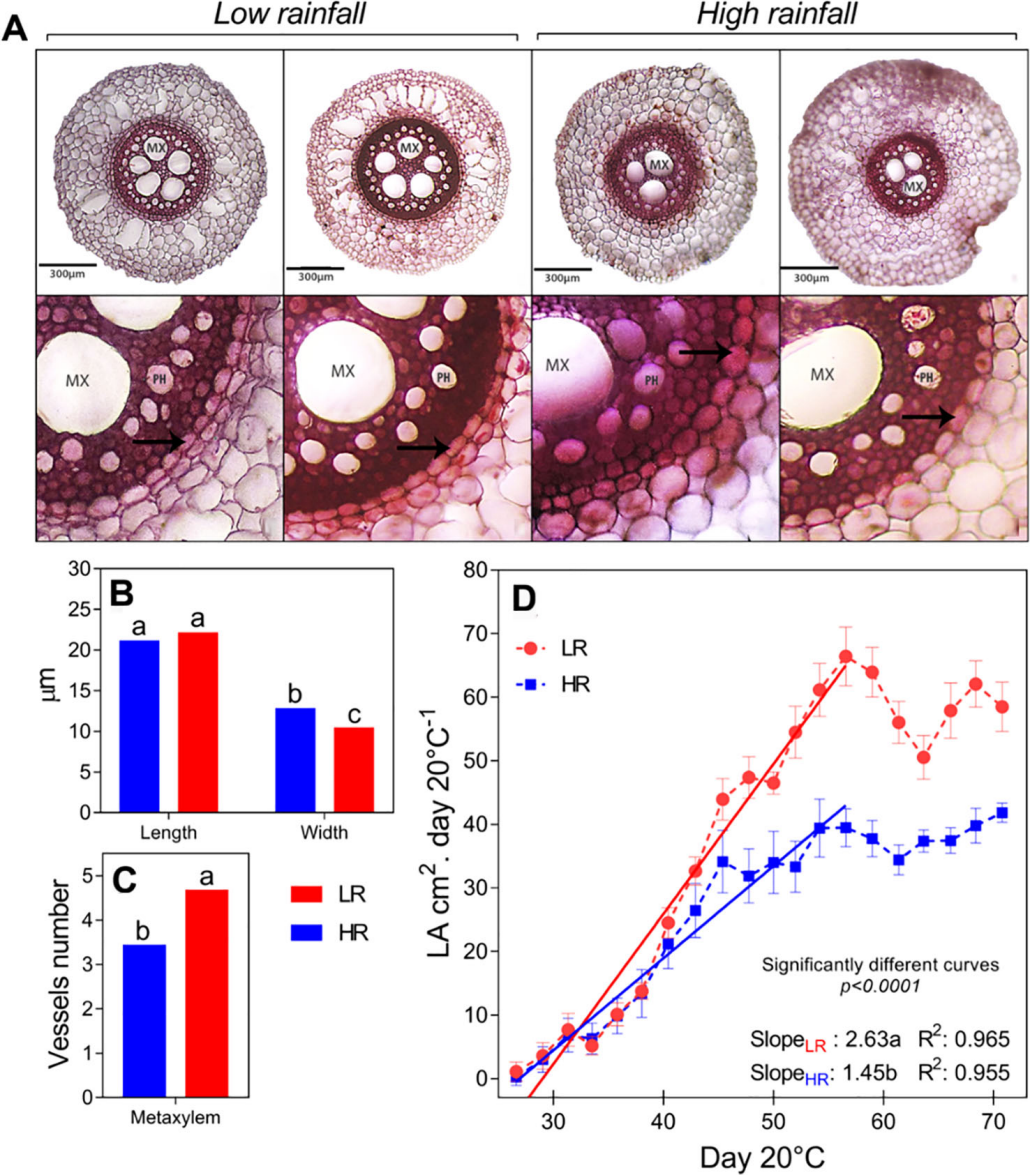
Root anatomical architecture and canopy growth dynamics. **(A)** Representative transverse cross-sections of root segments. Top row: Overview of roots from low rainfall (LR; HOPE 2013-AHT-R-14 and HOPE 2013-AHT-R-8) and high rainfall (HR; AHT-II/K13–24 and AHT-II/K13-5) genotypes. Bottom row: High-magnification view (10X objective) of a stele quadrant, highlighting the endodermal cell layer (arrow), metaxylem vessels (MX), and phloem (PH). **(B, C)** Quantitative anatomical analysis showing **(B)** mean endodermal cell size and **(C)** metaxylem vessel number. Different letters indicate significant differences between genotypes according to Fisher’s LSD test (p< 0.05). **(D)** Canopy development under high evaporative demand (Exp. 4). Curves display the time-course of leaf area expansion. Solid lines represent the linear regression model fitted to the exponential growth phase. Significant differences in growth rates **(slopes)** between HR and LR groups are indicated.

**Figure 5:**
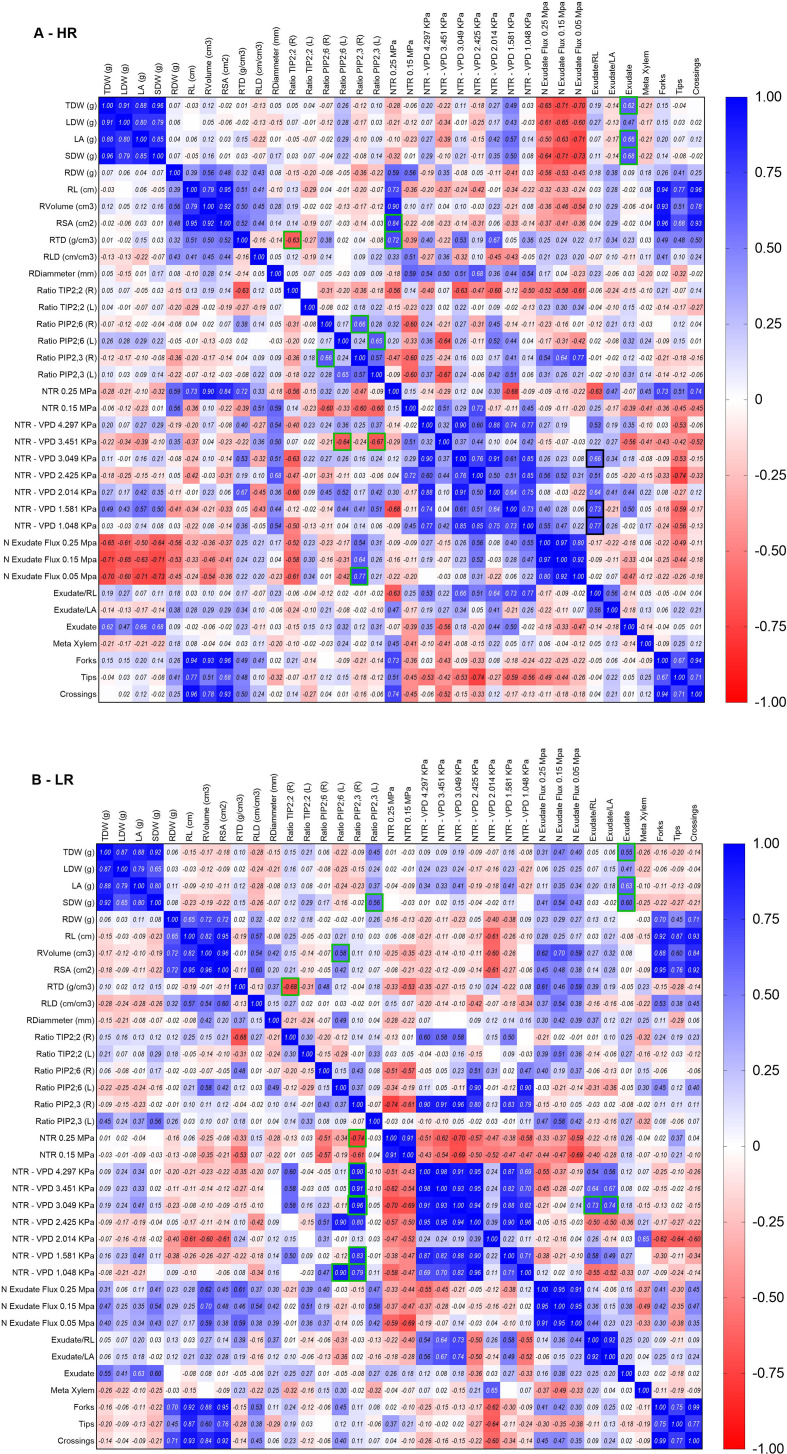
Integrative correlation analysis of aquaporin expression, root architecture, and transpiration response to VPD. **(A)** Overview of all traits measured across experiments conducted with high rainfall (HR; AHT-II/K13–24 and AHT-II/K13-5) genotypes, and **(B)** low rainfall (LR; HOPE 2013-AHT-R-14 and HOPE 2013-AHT-R-8) ones. Pearson correlation coefficients are shown from negative values (red) to positive values (blue), and green squares indicate highly significant correlations (p< 0.05). The analysis includes aquaporin expression and root exudate profiles.

Root phenotyping (Exp. 1 and Exp. 2) - LR hybrids consistently demonstrated a more robust root system than HR genotypes, regardless of the growth medium (hydroponics or soil/sand mixture). LR hybrids showed significantly higher root dry weight, total root length (RL), surface area (SA), Vroot volume, and root length per volume (**Table 1**). Detailed architectural analysis using WinRHIZO revealed that this expansion was largely driven by the proliferation of fine roots (diameter classes 0–2 mm) and a higher frequency of branching events (forks were higher in LR hybrids, specially between L and SA from 0 to 1 and V from 0 to 3) and root tips. Anatomical cross-sections (**Figure 4A** and **Table 1**) corroborated these macroscopic observations. LR hybrids displayed distinct vascular features, characterized by wider endodermal cells (**Figure 4A**) and a significantly higher number of metaxylem vessels (**Figures 4B, C**) compared to HR hybrids (**Figure 4A**). Moreover, the integrative correlation analysis (**Figure 5**) revealed significant relationships in LR genotypes, with Pearson coefficients ranging from 0.73 to 0.95 between PIP2;3 expression and the NTR response to VPD from 1 to 3.5 kPa, as well as with canopy growth (SDW) (r=0.56). PIP2;6 expression showed a correlation with root volume (r=0.57) and strong correlations with NTR under high VPD (r=0.89 at 2.4 kPa and r=0.90 at 1 kPa). TIP2;2 exhibited a correlation with root tissue density (RTD) (r=0.67). Similarly, root exudates were positively correlated (r=0.54–0.60) with TDW, LA, and SDW (**Figure 5B**). In addition, exudates per root length and per leaf area were highly correlated with the NTR response under high VPD (3 kPa). In HR genotypes, TIP2;2 showed a similar relationship with RTD (r=0.63), while PIP2;6 displayed a correlation with NTR under high VPD (3 kPa) (r=0.63). PIP2;3 was correlated with PIP2;6 (r=0.64–0.65), with NTR under high VPD (3 kPa), and with normalized exudation flow (r=0.76). Furthermore, exudates showed a correlation with TDW (r=0.66), LA, and SDW, and the exudation rate per root length was correlated with NTR across VPD levels from 1 to 3 kPa (**Figure 5B**).

**Table 1:** Morphometric root and shoot traits of pearl millet hybrids bred for high rainfall (HR) and low rainfall (LR) zones.

*Trait*	HR	LR	*p* value	*Trait*	HR	LR	*p* value
*Exp.2*				*Exp.1*			
*Aerial part*				*Aerial part*			
Leaf Area (cm^2^)	146	**217**	<0.000	Leaf Area (cm^2^)	118	157	ns
Stem dry weight (g)	0.45	**0.64**	<0.000	Stem dry weight (g)	0.22	**0.38**	0.039
Total dry weight (g)	1.44	**1.87**	<0.000	Total dry weight (g)	0.72	**1.07**	0.049
*Pressurized Roots*				*Roots*			
Root dry weight (g)	0.64	0.40	0.007	RLD *(cm.m^-3^)*	0.091	0.112	ns
Length (cm) L	4023	**5160**	0.03	RTD *(g.cm^-3^)*	1172	1084	ns
Surface Area (cm^2^) SA	429	**578**	0.02	***Roots treated with AgNO_3_***			
Average Diameter (mm)	0.34	0.36	ns	Root dry weight (g)	0.20	**0.31**	0.016
Volume (cm**^3^**) V	3.67	**5.18**	0.03	Volume>4	0.58	**1.19**	0.016
***RLD (cm.cm^-3^)***	1131	1007	ns	Surface Area>4	10	**23**	0.028
***Tips-T***	14794	**19505**	0.03	*Tips* *2<T<3*	0.5	**1.8**	0.013
***Forks***	22290	**36654**	0.09				
Fine roots (1–2 mm)				*Exp.3*			
**0<L ≤0.5**	3230	**4096**	0.03	***Aerial part***			
**0<L ≤1**	577	**736**	0.03	Leaf Area (cm^2^)	210	**317**	<0.000
***0<SA ≤ 0.5***	185	**237**	0.007	Stem dry weight (g)	0.40	**0.86**	<0.000
***0.5<SA ≤ 1***	128	**163**	0.01	Leaf dry weight (g)	0.55	**0.87**	<0.000
*0<V ≤0.5*	0.41	**0.49**	<0.000	Total dry weight (g)	0.94	**1.72**	<0.000
*0.5<V ≤1*	0.88	**1.03**	0.02				
*1<V ≤1.5*	0.68	**0.92**	0.01				
*1.5<V ≤2*	0.32	**0.46**	0.05				

Data represent the mean values of root architecture (length, surface Area, volume, branching) and biomass accumulation (root dry weight, stem dry weight) aggregated from Experiments 1 and 2. Differences between rainfall groups were assessed. Significance is indicated as follows: significant at p< 0.05; ns, not significant.Bold values mean LR values which are significantly higher than HR ones.

Aerial growth and canopy dynamics (Exp. 4) - To determine if these root hydraulic advantages translated to shoot performance under high evaporative demand, canopy development in vegetative stage was monitored outdoors using the LeasyScan platform. Leaf area (LA) of LR hybrids expanded significantly more rapidly than in HR hybrids (**Figure 4D**). Modeling of the exponential growth phase revealed a steeper slope for LR genotypes (2.63) compared to the HR group (1.45). Consistent with this canopy vigor, plant height (**Supplementary Figure 3**), stem dry weight, and total dry weight were significantly higher in LR- than in HR hybrids across all experimental setups (Exp. 1, 2, and 3; **Table 1**).

## 4 Discussion

In summary, this study uncovered a fundamental divergence in the hydraulic strategies of pearl millet hybrids adapted to contrasting agro-ecological zones. While pharmacological inhibition confirmed a generalized dependence on aquaporin (AQP)-mediated transport across all genotypes, the response to high VPD highlighted two distinct adaptive pathways. HR hybrids exhibited a conservative strategy, characterized by a higher constitutive specific root hydraulic conductivity that is rapidly restricted under stress, correlating with the drastic downregulation of root *PgPIP2;3* (**Figure 3C**). Conversely, LR hybrids display an opportunistic “spender” strategy. They compensate for a lower constitutive specific hydraulic conductivity via robust root anatomy and stable *PIP2;3* expression. This combination allowed LR hybrids to sustain high transpiration rates and rapid canopy expansion under high evaporative demand. Collectively, these findings suggest that adaptation to arid environments (zone A1) is not driven by strict water conservation, but by maximizing hydraulic capacity to support a robust growth when water is available and complete the life cycle before terminal drought sets in.

### 4.1 Inhibition of transpiration and the role of aquaporin-mediated transport

The significant reduction in transpiration observed across genotypes following HgCl_2_ and AgNO_3_ treatments confirms the pivotal role of aquaporins in maintaining whole-plant water status (**Figure 1**), aligning with reports in other cereals (Lopes et al., 2013; Liu et al., 2014). The ~25–40% decline in transpiration suggests that while the symplastic pathway is critical (Sadok and Sinclair, 2010), the apoplastic route remains a substantial component of the radial water transport.

A key genotypic divergence emerged regarding AgNO_3_ sensitivity. Unlike previous studies suggesting that high-transpiring genotypes of groundnut are insensitive to AgNO_3_ (Devi et al., 2012), our pearl millet LR hybrids were significantly inhibited (~40%). This implies that LR hybrids rely heavily on aquaporin-facilitated transport to sustain their “optimistic” water-spending strategy. Conversely, HR hybrids displayed higher xylem exudation under control conditions but strongly restricted transpiration under stress. This may suggests a high constitutive potential for radial uptake that is actively downregulated under high evaporative demand. Similar to coordinated mechanisms observed in tomato (Lucas Gutiérrez, 2025), HR hybrids likely employ a “hydraulic brake” to limit water loss, whereas LR hybrids maintain open hydraulic gates to maximize axial flow, a strategy probably supported by their vascular anatomy. Moreover, the lack of significant differences in Lpr when normalized with the root surface area between LR and HR controls, could be influenced by the methodological variability due to tissue disturbance during root extraction and anatomical barriers as reported in previous studies (Wang et al., 2023; Miyamoto et al., 2001, Miyamoto et al., 2001; Frensch and Steudle, 1989; Heymans et al., 2021). These factors may mask genotype-specific Lpr patterns.

### 4.2 Hydraulic conductance limitations and the radial transport constraint

Root pressurization revealed a delicate hydraulic balance: transpiration increased dramatically across all genotypes when root hydraulic resistance was alleviated. Crucially, this limitation was significantly more pronounced in LR hybrids, aligning with their lower constitutive root hydraulic conductivity.

An anatomical case emerges here: LR hybrids possess significantly numerous metaxylem vessels,which should theoretically lower axial resistance. Therefore, their primary hydraulic limitationmust reside within the radial pathway across the root cylinder. The smaller endodermal cells observed in LR hybrids probably imply a higher density of membrane barriers per unit length, potentially increasing this radial resistance. To compensate this radial hydraulic limitation under high VPD stress, LR hybrids exhibit a steeper transpiration response to increasing VPD (Suku et al., 2014), hypothesized to be driven by an inducible aquaporin-mediated pathway. Unlike HR hybrids, which hit a “hydraulic constraint,” LR hybrids actively upregulate *PIP2;3* in roots under high evaporative demand (Postaire et al. ,2010; Almeida-Rodriguez et al., 2011). This molecular upregulation likely facilitates a marked increase in the cell-to-cell water flow to overcome radial resistance (Barrios-Masias et al., 2015) and supply the large metaxylem vessels, mirroring adaptation strategies in drought-adapted grapevine and maize (Gambetta et al., 2012; Caldeira et al., 2014).

### 4.3 Transpiration dynamics and differential aquaporin regulation

Under high VPD, LR hybrids maintained significantly higher transpiration rates than HR hybrids, suggesting the latter actively restrict water loss. Our transcriptional profiling indicates this physiological divergence is likely governed by tissue-specific aquaporin regulation. While the pearl millet genome contains more than 20 aquaporin genes with probable functional redundancies (Grondin et al., 2020; Reddy et al., 2017), the differential regulation of *PgPIP2;3* serves as a potential strong correlative marker for these contrasting strategies.

In roots, HR hybrids significantly downregulated *PgPIP2;3* under high VPD. This repression potentially acts as a molecular “hydraulic brake,” limiting radial flow and triggering stomatal closure to conserve moisture, aligning with known water-saving phenotypes (Reddy et al., 2017). In contrast, LR hybrids maintained or slightly upregulated root *PIP2;3* expression. This molecular behavior likely supports a high-capacity transmembrane flow, matching the severe transpiration pull from the canopy at the cost of rapid soil water depletion.

In the shoot, physiological divergence relies on fine-tuned, antagonistic gating. LR hybridsmaintain stable *PgPIP2;3* expression and a baseline abundance of *PgPIP2;6* in leaves, facilitating high-capacity transcellular water flux to maximize carbon assimilation (Sakurai-Ishikawa et al., 2011; Zhang et al., 2013). Conversely, HR hybrids probably adopt a ‘safety-first’ strategy by actively recruiting *PgPIP2;6* in the shoots to restrict leaf hydraulic conductance and prevent xylem cavitation (Reddy et al., 2022). Furthermore, the slight upregulation of *TIP2;2* in both tissues (in HR leaves despite their lower transpiration) suggests an uncoupling of aquaporin expression from flux (Devi et al., 2016). This points to a potential prioritization of vacuolar osmotic adjustment (Xu et al., 2013). Consequently, these specific transcriptional patterns provide a hypothesis-generating framework, suggesting that the differential gating of these aquaporin isoforms may orchestrates the shift between acquisitive and conservative drought adaptations.

### 4.4 Functional coordination between root anatomy and canopy vigor

Plant growth dynamics depend on precise synchronization between root water uptake and canopy expansion. Our study reveals that LR hybrids achieved significantly greater biomass and leaf area expansion (**Figure 4D**, **Table 1**) compared to HR hybrids. This superior vegetative vigor supports the potential opportunistic strategy of maximizing carbon assimilation when water is available.

This canopy expansion is likely mechanically supported by a specialized root architecture (Richards and Passioura, 1989). While the overall root length density did not significantly differ between the groups (**Table 1**), LR roots exhibited a higher number of metaxylem vessels and thinner endodermal cells compared to HR genotypes. Defying trends seen in legumes where high vessel numbers are linked to wet environments (Purushothaman et al., 2013), this increased metaxylem number in LR hybrids suggests a priority for maximizing axial hydraulic capacity.

This anatomical “hardware” appears closely coupled with the molecular“software.” The sustained abundance of *PIP2;3* in LR roots likely serves to overcome radial resistance, supplying the high-capacity metaxylem vessels to match the transpiration demand and maintain leaf water potential of a rapidly expanding canopy (Maurel, 1997; Schoppach et al., 2014). This coordination aligns with the “functional hydraulic balance” hypothesis (Medina et al., 2017), where root hydraulic conductivity is fine-tuned to support shoot gas exchange. Thus, the LR phenotype may represents a coherent evolutionary design: a high-flow hydraulic system built to support rapid biomass accumulation, enabling swift lifecycle completion in erratic arid environments.

## 5 Conclusion

The breeding history of pearl millet, shaped by the contrasting predictability of rainfall in its adaptation zones, is distinctly reflected in the potential hydraulic strategies of modern hybrids. Our study reveals a clear functional divergence: hybrids bred for stable HR zones appear to adopt a “conservative” strategy characterized by hydraulic restriction under stress, whereas hybrids from rainfall-erratic LR zones may exhibit an “opportunistic” or acquisitive strategy when water is available.

Physiologically, LR hybrids could compensate for a lower constitutive root hydraulic conductivity through specialized anatomical features like a higher network of metaxylem vessels that, despite being slightly narrower, maximize total axial transport capacity. Under high evaporative demand, these hybrids demonstrate a superior ability to increase transpiration. We propose that this physiological plasticity could be driven by a coordinated molecular valve mechanism. Specifically, the sustained expression or upregulation of *PgPIP2;3* in the roots of LR hybrids which contrast with its massive downregulation in HR genotypes, that facilitates rapid cell-to-cell water flow across the root cylinder.

This aquaporin-mediated pathway could effectively “unlocks” the high axial capacity of their highly vascularized root network. This aggressive hydraulic coordination supports the superior biomass accumulation and canopy expansion observed in LR genotypes, enabling them to maximize productivity and complete their lifecycle rapidly during transient periods of water availability in arid environments.

## Data Availability

The datasets presented in this study can be found in online repositories. The names of the repository/repositories and accession number(s) can be found in the article/**Supplementary Material**.
