# Supplementary material for: Water flux patterns and aquaporin dynamics: linking stomatal demand to root hydraulics in pearl millet hybrids
Source: Front Plant Sci. 2026 Apr 14;17:1776229. doi: 10.3389/fpls.2026.1776229 (PMC13122772; doi:10.3389/fpls.2026.1776229)
Supplement: Supplementary file 1 [file DataSheet1.docx]

Supplementary Material

# Supplementary Figures and Tables

## Supplementary Figures

**Supplementary Figure 1. Geographical location of high (HR) and low (LR) rainfall zones in India**. Selected 62 districts representing 90% of the total kharif pearl millet area over the period 1998–2017. The districts are coloured according to the reference rain and geography criteria considering in the Northern part the LR zone (A1: < 400 mm/year in red), the A zone > 400 mm/year in orange, and the HR zone in the south > 400 mm/year in yellow. Adapted from Garin et al., 2023.

**
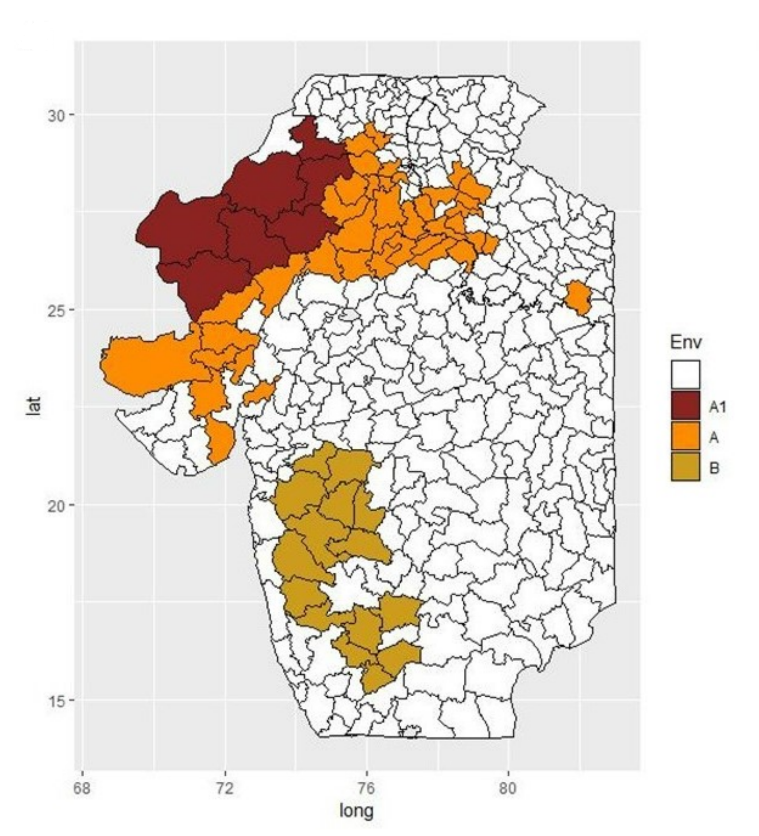
**

**Supplementary Figure 2. Pharmacological aquaporin inhibition in plant transpiration higher and lower rainfall genotypes.** Normalized Transpiration Rate (NTR) kinetics following treatment with the aquaporin blockers **(triangle)** mercuric chloride (HgCl_2_) and **(square)** silver nitrate (AgNO_3_). The magnitude of inhibition relative to the non-treated plants baseline (green line) was quantified using Area Under the Curve (AUC) analysis. Data represent means (of 5 replicates per treatment and genotype) of High Rainfall (HR in blue) and Low Rainfall (LR in red) genotypes.

**
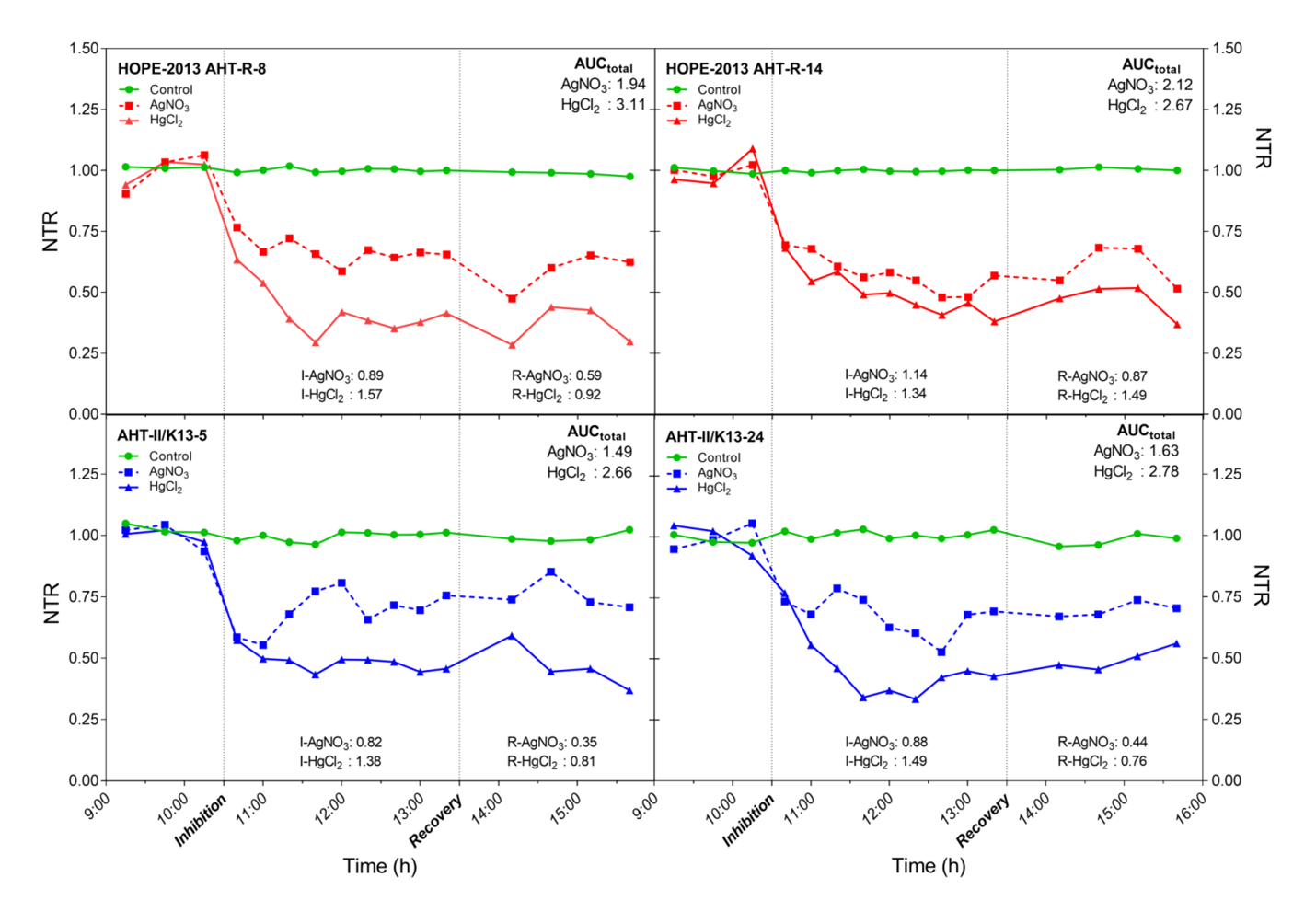
**

**Supplementary figure 3. Canopy growth dynamics.** Canopy development under high evaporative demand (Exp. 4) of High Rainfall (HR) and Low Rainfull (LR) groups of genotypes. Curves display the time-course of Leaf Area (LA) in expansion (A), exponential growth phase in vegetative stage where solid lines represent the linear regression model fitted (B) and projected leaf area (PLA) as well as Plant height (D). Significant differences in growth rates (slopes) between HR and LR groups are indicated.

**
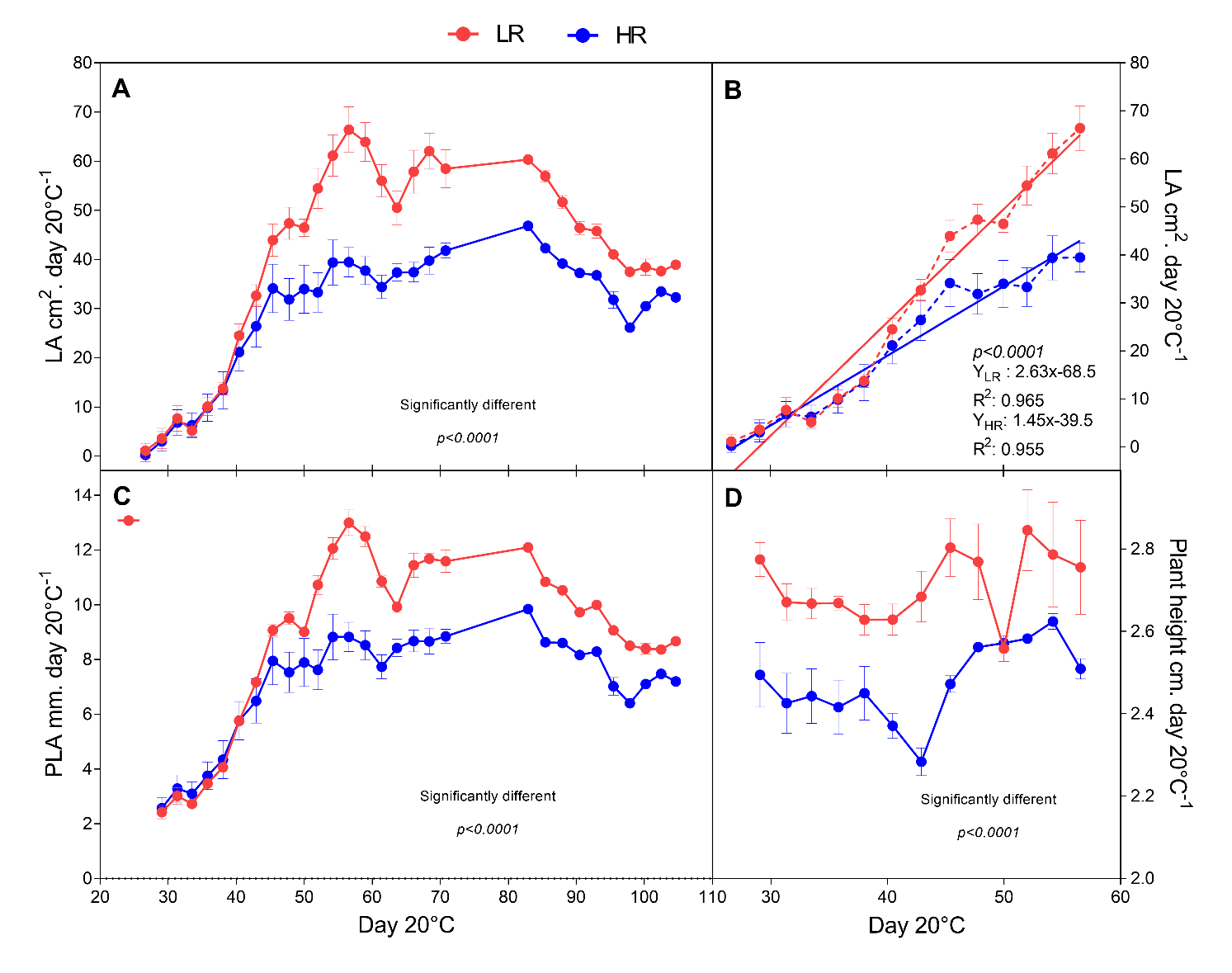
**

## Supplementary Tables

**Supplementary Table 1. Primes used in RT-qPCR assay.** Protein ID and accession number are indicated, details are described elsewhere (Reddy et al., 2015a, 2015b)

| Primer  (Accession) | Protein name | Sequence  F: forward and R: reverse |
| --- | --- | --- |
| qPgPIP2;3  (XP_002461931) | Plasma membrane intrinsic protein | F: GTTCGCGGTTTTCATGGTC  R: AGAAGATCCGGTGGTCATCC |
| qPgPIP2;6  (XP_002461936) | Plasma membrane intrinsic protein | F: GTGATCGGGTACAAGCACCA  R: CGGTGCAGTAGACGAGGATG |
| qPgTIP2;2  (XP_002438430) | Tonoplast intrinsic protein | F: CTCCCTCAGGGCCTACGTC  R: GCCGTCGCTCAACTTTCTG |
| qPgEF-1α  (EF694165) | Elongation factor 1-alpha | F: AATGATCCGCTGCTGTAACAAG  R: AGGCAATCTTGTCTGGGTTGTA |
| qPgEIF4A  (EU856535) | Eukaryotic initiation factor 4A | F: ACTGAAAGAATGCGCAGCAA  R: ACGAGTTGCACCAGACCTGA |
| qPgACP  (KM105958) | Acyl carrier protein | F: AGCAACCAGTGCCACAAAGA  R: GGAACTTGGAGGAGCCAGAA |

**Supplementary Table 2. Aquaporin fold change variation in leaf and root tissues.** Fold change shows the variation in expression of PIP 2;3. PIP2;6 and TIP 2;2 within afternoon (T1 and T2) and morning (C) expression (upper panel), low (LR) and high (HR) rainfall zones (mid panel) and high and low VPD (bottom panel) expressed as a log_2_ transformation of 2^-∆∆Ct^, significant differences were assessed with LSD test (ns, *non-significant*; *,p<0.05; **,p<0.01 and ***,p<0.001). For experiment design see figure 3.

| Fold change in expression | **PIP 2;3** | | **PIP 2;6** | | **TIP 2;2** | |
| --- | --- | --- | --- | --- | --- | --- |
| Condition | Leaf | Root | Leaf | Root | Leaf | Root |
| *Afternoon T2 respect to morning C.* | | |  |  |  |  |
| HR at ladder of VPD | -3.30*** | -14.68*** | -1.10 ns | -1.47** | 1.04 ns | -1.18*** |
| HR at constant low VPD | -2.10 *** | -2.073* | -2.86*** | -1.06 ns | -1.74*** | -1.17 ns |
| LR at ladder of VPD | -4.98*** | -2.05 ns | -1.37* | -1.01 ns | 1.10 ns | -1.08 ns |
| LR at constant low VPD | -2.69**** | 1.46** | 4.99* | 1.29** | 3.62** | 1.35* |
|  |  |  |  |  |  |  |
| *Low rainfall respect to high rainfall.* | | |  |  |  |  |
| Ladder of VPD-T2 (high VPD) | **1.14*** | **4.94 ns** | **1.22 ns** | **1.22 ns** | **-1.05 ns** | **-1.05 ns** |
| Ladder of VPD-C (low VPD) | 1.72 ns | -1.45 ns | 1.52* | -1.19 * | -1.12 ns | -1.15 ns |
| Constant low VPD-T1 | **1.74*** | **1.46*** | **-1.01 ns** | **1.24**** | **1.49*** | **1.04 ns** |
| Constant low VPD-C | 2.03* | -2.07 ns | -14.45*** | -1.11 ns | -4.22** | -1.52** |
|  |  |  |  |  |  |  |
| *High VPD respect to low VPD* | | |  |  |  |  |
| HR on the afternoon T2 | **1.36 ***** | **-6.43 ns** | **2.03**** | **-1.21 ns** | **1.77***** | **-1.45*** |
| LR on the afternoon T1 | **-1.12 ns** | **1.13*** | **2.52***** | **-1.23**** | **1.12 ns** | **-1.59***** |
| HR on the morning C | 1.95* | 1.10 ns | -1.28 ns | 1.14 ns | -1.02 ns | -1.44* |
| LR on the morning C | 1.65 ns | 1.57 ns | 17.23* | 1.06 ns | 3.66* | -1.09 ns |

# Note: This research is derived from the doctoral thesis titled 'Differential responses of historical cereal lines to water stress: phenotype and gene expression' (Respuesta diferencial de líneas históricas de cereales frente al estrés hídrico: fenotipo y expresión genómica), available at the University of Barcelona repository ([https://hdl.handle.net/2445/117908](https://www.google.com/search?q=https://hdl.handle.net/2445/117908)). All relevant data are contained within this article and the repository. Any additional raw data supporting the conclusions will be made available by the authors, without undue reservation.
